# Immune Modulation of NYVAC-Based HIV Vaccines by Combined Deletion of Viral Genes that Act on Several Signalling Pathways

**DOI:** 10.3390/v10010007

**Published:** 2017-12-27

**Authors:** Carmen Elena Gómez, Beatriz Perdiguero, Cristina Sánchez-Corzo, Carlos Oscar S. Sorzano, Mariano Esteban

**Affiliations:** 1 Department of Molecular and Cellular Biology, Centro Nacional de Biotecnología, Consejo Superior de Investigaciones Científicas (CNB-CSIC), Campus de Cantoblanco, 28049 Madrid, Spain; cegomez@cnb.csic.es (C.E.G.); perdigue@cnb.csic.es (B.P.); cscorzo@cnb.csic.es (C.S.-C.); 2 Biocomputing Unit, Centro Nacional de Biotecnología, Consejo Superior de Investigaciones Científicas (CNB-CSIC), Campus de Cantoblanco, 28049 Madrid, Spain; coss@cnb.csic.es

**Keywords:** HIV-1, NYVAC, immunogenicity, T and B cell immune response, Toll-like receptor, interferon, immunomodulators, cytokines/chemokines

## Abstract

An HIV-1 vaccine continues to be a major target to halt the AIDS pandemic. The limited efficacy of the RV144 phase III clinical trial with the canarypox virus-based vector ALVAC and a gp120 protein component led to the conclusion that improved immune responses to HIV antigens are needed for a more effective vaccine. In non-human primates, the New York vaccinia virus (NYVAC) poxvirus vector has a broader immunogenicity profile than ALVAC and has been tested in clinical trials. We therefore analysed the HIV immune advantage of NYVAC after removing viral genes that act on several signalling pathways (Toll-like receptors—TLR—interferon, cytokines/chemokines), as well as genes of unknown immune function. We generated a series of NYVAC deletion mutants and studied immune behaviour (T and B cell) to HIV antigens and to the NYVAC vector in mice. Our results showed that combined deletion of selected vaccinia virus (VACV) genes is a valuable strategy for improving the immunogenicity of NYVAC-based vaccine candidates. These immune responses were differentially modulated, positive or negative, depending on the combination of gene deletions. The deletions also led to enhanced antigen- or vector-specific cellular and humoral responses. These findings will facilitate the development of optimal NYVAC-based vaccines for HIV and other diseases.

## 1. Introduction

UNAIDS (Joint United Nations Programme on HIV/AIDS) estimates that at the end of 2016, 36.7 million people worldwide lived with HIV (www.unaids.org); the number of newly infected individuals nonetheless continues to fall as the result of global implementation of preventive and therapeutic strategies. Vaccine development remains among the best hopes for controlling the HIV/AIDS pandemic.

To date, RV144 is the only HIV-1 vaccine efficacy trial that demonstrated a modest protection level (31%) [1]. This study combined a recombinant canarypox vector (ALVAC-HIV, vCP1521) as a prime component, with a recombinant HIV-1 envelope gp120 protein (AIDSVAX B/E) as a boost. Further studies seeking immune correlates of protection showed that non-neutralizing antibodies to HIV-1 Env V1/V2 regions were associated with reduced risk of HIV-1 acquisition, whereas IgA antibodies to the envelope correlated with decreased vaccine efficacy [2,3]. High levels of antibody-dependent cell-mediated cytotoxicity (ADCC) also correlated with reduced risk of infection [4]. These clinical findings provided evidence that an HIV/AIDS vaccine can prevent HIV-1 infection and highlighted the potential of replication-deficient live recombinant viral vectors and heterologous prime-boost regimes to elicit protective immune responses. Current HIV vaccine development efforts thus focus on optimizing such priming and boosting components.

As an alternative to the ALVAC vector, the highly attenuated vaccinia virus (VACV) strain NYVAC [5] is a potential poxvirus-based HIV vaccine candidate. NYVAC-based recombinants have been evaluated in preclinical [6,7,8,9] and clinical studies [10,11,12], with encouraging results. In non-human primates, the NYVAC vector was superior to ALVAC in inducing HIV immunity [13]. New strategies are thus being implemented to improve NYVAC vector immunogenicity.

In mice, we were able to improve HIV-1-specific immune responses to the NYVAC-C recombinant vector expressing HIV-1 antigens by deletion of immunomodulatory genes such as *B8R* and/or *B19R* (which block type II and type I interferon—IFN—signalling pathways, respectively) [14], by single deletion of the VACV-TLR inhibitor *A46R* [15], or by single, double or triple deletion of VACV-TLR inhibitors *A52R*, *K7R* and *B15R* [16,17].

To further describe the role of viral genes in NYVAC vector immunogenic potential, we sought to define in mice the effect of deleting from the NYVAC-C genome various combinations of viral genes that inhibit TLR, IFN and cytokine/chemokine host-cell antiviral pathways, as well as some unknown non-essential genes that accompanied *B8R*. We thus characterized HIV- and VACV-specific CD4 and CD8 T cells and antibody levels in mice immunized with these NYVAC deletion mutants. The deletions modulated HIV-1- and VACV-specific responses distinctly and some provided more immune enhancement than others. These results demonstrate that NYVAC-based vaccine immunogenicity can be regulated by genetic modification of the parental vector, which could extend use of these vectors for vaccine development.

## 2. Materials and Methods

### 2.1. Ethics Statement

Animal experimental protocols were approved by the Ethical Committee of Animal Experimentation (CEEA-CNB) of Centro Nacional de Biotecnologia (CNB-CSIC, Madrid, Spain) in accordance with Spanish National Royal Decree (RD 1201/2005), International EU Guidelines 2010/63/UE on protection of animals used for experimentation and other scientific purposes and Spanish National Law 32/2007 on animal welfare and their exploitation, transport and sacrifice (permit numbers 10-018, 10-023, 152/07 and 080030).

### 2.2. Cells and Viruses

African green monkey kidney cells (BSC-40) and primary chicken embryo fibroblasts (CEF) were grown in Dulbecco’s modified Eagle’s medium (DMEM) supplemented with 100 U/mL penicillin, 100 μg/mL streptomycin (both from Invitrogen, Carlsbad, CA, USA), 2 mM l-glutamine (Merck, Kenilworth, NJ, USA) and 10% new-born calf serum (NCS; Sigma, St. Louis, MO, USA) for BSC-40 cells or 10% foetal calf serum (FCS; Sigma) for CEF. Cells were maintained in humidified air with 5% CO_2_ at 37 °C. Poxvirus strains used included the genetically attenuated vaccinia-based vector NYVAC-WT (vP866; provided by Sanofi-Pasteur, Swiftwater, PA, USA), the recombinant NYVAC-C expressing gp120 as a cell released product and Gag-Pol-Nef from the clade C CN54 HIV-1 isolate as an intracellular polyprotein [8], as well as the NYVAC-C-based deletion mutants NYVAC-C-∆A46R [15] (here termed NYVAC-C-∆TLR1), NYVAC-C-∆B19R [18] and NYVAC-C-∆B8R/∆B19R [14], used here as parental vectors to generate the following NYVAC-based deletion mutants: NYVAC-C-∆A46R/∆A52R (NYVAC-C-∆TLR2), NYVAC-C-∆A46R/∆A52R/∆K7R (NYVAC-C-∆TLR3), NYVAC-C-∆A46R/∆A52R/∆K7R/∆B15R (NYVAC-C-∆TLR4) and NYVAC-C-∆B19R/∆B6R-B10R (Table 1). All viruses were grown in CEF, purified through two 36% (w/v) sucrose cushions and titrated by immunostaining in a plaque assay in BSC-40 cells as described [19]. All viruses were titrated at least three times. After all infections, complete DMEM supplemented with 2% NCS or FCS was added to cultured cells.

### 2.3. Construction of the Plasmid Transfer Vector pGem-RG-B6R-B10R-wm

The plasmid transfer vector pGem-RG-B6R-B10R-wm, used for deletion of *B6R-B10R* open reading frames (ORFs) from the NYVAC-C genome, was obtained by sequential cloning of *B6R* and *B10R* recombination flanking sequences into the plasmid pGem-Red-GFP wm [18]. The NYVAC genome was used as template to amplify the left flank of the *B6R* gene with oligonucleotides LFB6R-AatII-F (5′-GGAATGACGTCCTCCCAATATGTG-3′) (AatII site underlined) and LFB6R-XbaI-R (5′-GCTCTAGACTCAATTCATTCTAGC-3′) (XbaI site underlined). The left flank was digested with AatII and XbaI and cloned into plasmid pGem-Red-GFP wm previously digested with the same restriction enzymes to generate pGem-RG-LFsB6R wm (4881 bp). The right flank of the *B10R* gene was amplified by PCR from the NYVAC genome with oligonucleotides RFB10R-ClaI-F (5′-CCATCGATTTGAAAATGAAAATATAAATAG-3′) (ClaI site underlined) and RFB10R-BamHI-R (5′-CGGGATCCAGTAGATATGATCTATATTC-3′) (BamHI site underlined), digested with ClaI and BamHI and inserted into the ClaI/BamHI-digested pGem-RG-LFsB6R wm to generate pGem-RG-LFsB6R-RFB10R wm (5225 bp). The repeated left flank of the *B6R* gene was amplified by PCR from the NYVAC genome with oligonucleotides LFB6R′-EcoRI-F (5′-CGGAATTCCTCCCAATATGTGTACG-3′) (EcoRI site underlined) and LFB6R′-ClaI-R (5′-CCATCGATCTCAATTGATTCTAGC-3′) (ClaI site underlined), digested with EcoRI and ClaI and inserted into the EcoRI/ClaI-digested pGem-RG-LFsB6R-RFB10R wm. The resulting plasmid, pGem-RG-B6R-B10R-wm (5558 bp), was confirmed by DNA sequence analysis and directs deletion of the *B6R-B10R* cassette from the NYVAC genome.

The plasmid transfer vectors pGem-RG-A52R-wm, pGem-RG-K7R-wm and pGem-RG-B15R-wm, used to delete *A52R*, *K7R* and *B15R* ORFs from the NYVAC-C genome, respectively, were obtained by the same method and have been reported [17].

### 2.4. Construction of NYVAC-Based Deletion Mutants

The different NYVAC-based deletion mutants generated and the corresponding parental viruses and plasmid transfer vectors used in the infection/transfection protocol are listed in Table 1. NYVAC-based deletion mutants were constructed using dsRed2 and rsGFP markers. BSC-40 cells (3 × 10^6^) were infected with 0.005 pfu (plaque-forming units)/cell of parental virus and transfected 1 h later with 6 μg DNA of specific plasmid transfer vector using Lipofectamine (Invitrogen; Thermo Scientific Inc., USA). At 72 h post-infection, cells were harvested, lysed by freeze-thaw cycling, sonicated and used for recombinant virus screening. Deletion mutants were selected from progeny virus by consecutive rounds of plaque purification in BSC-40 cells, during which plaques were screened for Red2/GFP fluorescence. In the first three passages, viruses from selected plaques expressed both fluorescent proteins; in the next two passages, viral progeny from selected plaques expressed only one fluorescent marker. In the last two passages (seven passages total), viruses from selected plaques did not express a fluorescent marker due to marker loss by homologous recombination within the repeated flanking DNA sequences.

### 2.5. PCR Analysis of Deletion Mutants

To test for correct generation and purity of the deletion mutants, viral DNA was extracted from BSC-40 cells infected at 5 pfu/cell with NYVAC-WT, NYVAC-C, or the different NYVAC-C deletion mutants. Cell membranes were disrupted by proteinase K treatment (0.2 mg/mL proteinase K in 50 mM Tris-HCl pH 8, 100 mM EDTA (ethylenediaminetetraacetic acid) pH 8, 100 mM NaCl, 1% SDS; 1 h, 55 °C), followed by incubation with RNase A (80 μg/mL). Viral DNA was precipitated using 2-propanol. Different sets of primers annealing in the gene-flanking regions to be deleted were used for PCR analysis of the loci. The amplification reactions were carried out with Phusion High-Fidelity DNA polymerase (BioLabs, Ipswich, MA, USA). Primers used and size of the expected PCR products are shown in Table 2.

### 2.6. Western Blot Detection of HIV-1 Protein gp120 and Gag-Pol-Nef Expression

To test correct expression of HIV-1 antigens by the deletion mutants, monolayers of BSC-40 cells were infected at 5 pfu/cell with NYVAC-WT, NYVAC-C, or the different NYVAC-C deletion mutants. At 24 h post-infection, cells were lysed in Laemmli buffer; cell extracts were fractionated by 8% SDS-PAGE and analysed by Western blot using rabbit polyclonal anti-gp120 antibody (1:3000; Centro Nacional de Biotecnología, Madrid, Spain) or rabbit polyclonal anti-gag p24 serum (1:1000; ARP 432, NIBSC Centralised Facility for AIDS Reagents, South Mimms, Harts, UK) to evaluate gp120 and Gag-Pol-Nef (GPN) protein expression, respectively. Anti-rabbit-horseradish peroxidase (1:5000; Sigma) was used as secondary antibody. Immune complexes were detected by enhanced chemiluminescence (ECL, GE Healthcare, Little Chalfont, UK).

### 2.7. Analysis of Virus Growth

To determine virus growth profiles, CEF monolayers grown in 12-well tissue culture plates were infected at 0.01 pfu/cell with NYVAC-WT, NYVAC-C, or the different NYVAC-C deletion mutants. Following virus adsorption (60 min, 37 °C), the inoculum was removed. Infected cells were washed once with serum-free DMEM and incubated with fresh DMEM containing 2% FCS (37 °C, 5% CO_2_). At different times post-infection (0, 24, 48 and 72 h), cells were removed by scraping and freeze-thawed three times; lysates were prepared from 5 × 10^5^ cells/mL and briefly sonicated. Virus titres in cell lysates were determined by immunostaining in a plaque assay in BSC-40 cells, as described [19].

### 2.8. DNA Vectors 

The two DNA constructs expressing the HIV-1 _CN54_gp120 (pcDNA-_CN54_gp120) and HIV-1 _CN54_Gag-Pol-Nef (GPN) fusion protein (pcDNA-_CN54_GPN) have been reported [8]. Plasmids were purified using the Maxiprep purification kit (Qiagen) and diluted for injection in endotoxin-free phosphate-buffered saline (PBS).

### 2.9. Peptides

The HIV-1 peptide pools Env-1, Env-2, Gag-1, Gag-2, GPN-1, GPN-2, GPN-3 and NEF were provided by the EuroVacc Foundation (Lausanne, Switzerland) and have been described [8]. They spanned the HIV-1 Env, Gag, Pol and Nef antigens from clade C included in the immunogens as consecutive 15-mers overlapping by 11 amino acids. For analysis of HIV-1-specific cellular immune responses, we grouped the pools as follows: Env pool (Env-1 + Env-2), Gag pool (Gag-1 + Gag-2) and GPN pool (GPN-1 + GPN-2 + GPN-3 + NEF). The VACV peptide E3_140–148_ (VGPSNSPTF; CNB-CSIC Proteomics Service), described as an immunodominant epitope in BALB/c mice [29], was used to detect VACV-specific CD8 T cell responses.

### 2.10. Mouse Immunization Schedule

BALB/c mice were purchased from Harlan. The DNA prime/poxvirus boost immunization protocol was used to assay the immunogenicity of the deletion mutants. Groups of 6- to 8-week-old female mice (*n* = 4) received 100 μg DNA-C (50 μg pcDNA-_CN54_gp120 + 50 μg pcDNA-_CN54_GPN) by the intramuscular route (i.m.); two weeks later, they received an intraperitoneal (i.p.) inoculation of 1 × 10^7^ pfu of the corresponding virus. The control group was primed with sham DNA (DNA-ϕ) and boosted with non-recombinant NYVAC-WT. At 53 days after the last immunization (memory phase), mice were sacrificed and spleens and sera were processed for intracellular cytokine staining (ICS) and Enzyme-Linked ImmunoSorbent (ELISA) assays to analyse cellular and humoral immune responses to HIV-1 and VACV antigens, respectively. Representative data are shown for 2–3 experiments.

### 2.11. Intracellular Cytokine Staining Assay (ICS)

The magnitude and phenotype of HIV- or VACV-specific T cell responses were analysed by ICS. After an overnight rest, 4 × 10^6^ splenocytes (erythrocyte-depleted) were seeded on 96-well plates and stimulated (6 h) in complete Roswell Park Memorial Institute (RPMI) 1640 medium with 10% FCS, 1 μL/mL Golgiplug (BD Biosciences, San Jose, CA, USA) and 1 μg/mL of the different HIV-1 peptide pools or 10 μg/mL of E3 peptide. After stimulation, cells were washed, stained for surface markers, permeabilized (Cytofix/Cytoperm kit; BD Biosciences) and stained intracellularly using appropriate fluorochromes. Fluorochrome-conjugated antibodies were used for functional analyses (CD4-Alexa 700, CD8-FITC or -V500, IL-2-APC, IFN-γ-PeCy7, TNF-α-PE) and for phenotypic analyses (CD62L-FITC, CD44-SPRD). Dead cells were excluded using the violet LIVE/DEAD stain kit (Invitrogen). All antibodies were from BD Biosciences. Cells were acquired on an LSRII flow cytometer (BD Immunocytometry Systems, Franklin Lakes, NJ, USA). Data analyses were performed using FlowJo software v.8.5.3 (TreeStar, Ashland, OR, USA). The number of lymphocyte-gated events ranged between 10^5^ and 10^6^. After gating, Boolean combinations of single functional gates were generated using FlowJo to determine the frequency of each response, based on all possible combinations of cytokine expression or of differentiation marker expression. Background responses in negative control samples were subtracted from those in stimulated samples for each functional combination. Graphs were generated using GraphPad software (Version 6.01, San Diego, CA, USA).

### 2.12. Antibody Measurement by ELISA

Antibody binding to Env and vaccinia virus proteins in serum was assessed by ELISA as described [8]. Sera from naïve and immunized mice were diluted serially (3-fold) in duplicate and incubated with 2 μg/mL recombinant CN54gp120 purified protein (ARP683, HIV-1 CN54gp120 clade C; EU Programme EVA, NIBSC Centralised Facility for AIDS Reagents) or 10 μg/mL extract of BSC-40 cells infected (5 pfu/cell) for 24 h with VACV WR (Western Reserve) strain. Antibody titres of Env- or VACV-specific IgG were defined as the last serum dilution that gave three times the mean OD_450_ value of the naïve control.

### 2.13. Plaque Neutralization Assay

To measure the neutralizing titre of anti-VACV antibodies, vaccinated mice were exsanguinated and sera were prepared and heated at 56 °C for 30 min to inactivate complement. Two-fold dilutions of serum in DMEM (Gibco, Waltham, MA, USA) supplemented with 2% NCS were prepared and incubated with about 400 pfu of purified VACV WR for 1 h at 37 °C before plaque assay on BSC-40 cells grown in 6-well plates. After 48 h of incubation at 37 °C, plaques were visualized by staining with 1% crystal violet in 2% ethanol. ND_50_ (Neutralization dose 50) values represent the reciprocal of the serum dilution giving 50% reduction in plaque number compared with virus incubated without serum.

### 2.14. Murine Intranasal Challenge

Female BALB/c mice (*n* = 5, 6–8 weeks old) were infected intraperitoneally (i.p.) with 10^7^ pfu of NYVAC-WT, NYVAC-C or NYVAC-C deletion mutants in TLR immunomodulatory genes. PBS-treated mice were used as control group. One month later, animals were challenged intranasally (i.n.) with 5 × 10^6^ pfu of wild-type VACV WR. The weight loss was monitored daily for 15 days.

### 2.15. Data Analysis and Statistics

For statistical analysis of ICS data, we corrected measurements for the unstimulated control sample response (RPMI) and calculated confidence intervals and *p* values of hypothesis tests [30,31]. Only antigen response values significantly higher than the RPMI value are represented; background for the distinct cytokines in unstimulated controls were never >0.05%. For statistical analysis of the humoral response measured by ELISA, two-way ANOVA multiple comparison was used.

## 3. Results

### 3.1. Generation and In Vitro Characterization of NYVAC-C Deletion Mutants

**Figure 1:**
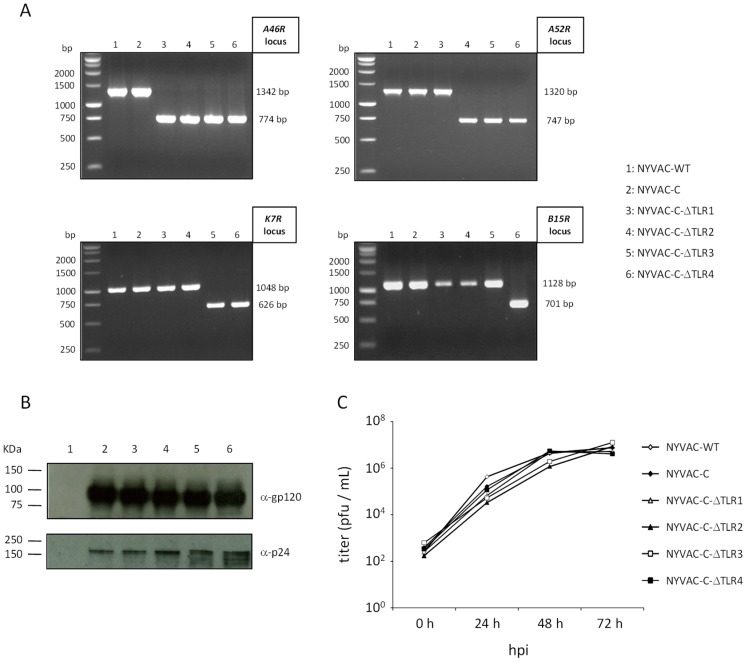
In vitro characterization of NYVAC-C deletion mutants involved in toll-like receptor (TLR) signalling inhibition. (**A**) Confirmation of *A46R*, *A52R*, *K7R* and *B15R* gene deletion by PCR analysis. Viral DNA was extracted from BSC-40 cells infected with NYVAC-WT, NYVAC-C or NYVAC-C deletion mutants (5 pfu/cell). Primers spanning gene flanking sequences were used for PCR analysis of *A46R*, *A52R*, *K7R* and *B15R* loci. Sizes obtained in parental NYVAC and in deletion mutants are indicated. (**B**) Western blot showing HIV antigen expression. BSC-40 cells were infected as in (**A**). At 24 h post-infection, cells were lysed in Laemmli buffer, cell extracts were fractionated by 8% SDS-PAGE and analysed in Western blot using polyclonal anti-gp120 antibody or anti-gag p24 serum to evaluate gp120 (120 kDa) and GPN (150 kDa) expression, respectively. (**C**) Analysis of virus growth in CEF cells. CEF cell monolayers were infected with NYVAC-WT, NYVAC-C or NYVAC-C-ΔTLR1-4 (0.01 pfu/cell). At various times post-infection (0, 24, 48 and 72 h), cells were collected and infectious viruses quantified by immunostaining plaque assay in BSC-40 cells.

**Figure 2:**
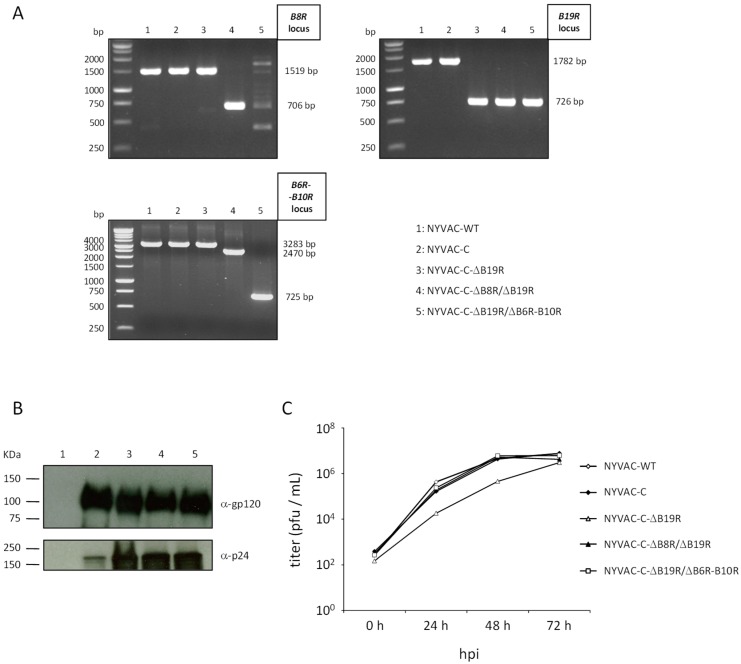
In vitro characterization of NYVAC-C mutants with deletion of genes involved in interferon (IFN) and cytokine signalling inhibition. (**A**) Confirmation of *B8R*, *B19R* and *B6R-B10R* gene deletion by PCR analysis. Viral DNA was extracted from BSC-40 cells infected as in Figure 1A. Primers spanning gene flanking sequences were used for PCR analysis of *B8R*, *B19R* and *B6R-B10R* loci. Sizes for parental NYVAC and deletion mutants are indicated. (**B**) Western blot showing HIV antigen expression. BSC-40 cells were infected as in Figure 1A. At 24 h post-infection, cells were lysed and cell extracts fractionated and analysed as in Figure 1B. (**C**) Analysis of virus growth in CEF cells. CEF cell monolayers were infected with NYVAC-WT, NYVAC-C or NYVAC-C deletion mutants (0.01 pfu/cell). At various times post-infection (0, 24, 48. and 72 h), cells were collected and infectious viruses quantified by immunostaining plaque assay in BSC-40 cells.

All NYVAC-C-based deletion mutants used and the site of action of the deleted genes are detailed in Table 1. They were generated as described (see Section 2) using as parental virus the NYVAC-C recombinant or NYVAC-C-based deletion mutants that express HIV-1 Env and GPN antigens from clade C [8]. Correct gene deletion was confirmed by PCR using primers that annealed in gene-flanking sequences. The distinct viral genes were deleted correctly, with no wild-type contamination in NYVAC preparations (Figure 1A, deletion of VACV-TLR inhibitors *A46R*, *A52R*, *K7R* and *B15R*; Figure 2A, deletion of VACV-cytokine/chemokine inhibitors *B8R* and/or *B19R* and/or *B6R-B10R* cassette). Western blot analysis indicated that NYVAC-C-based deletion mutants expressed HIV-1 gp120 (120 kDa) and GPN (150 KDa) proteins at levels similar to the parental NYVAC-C virus (Figure 1B and Figure 2B). To determine whether deletion of specific viral genes affected virus replication, we compared virus growth kinetics of NYVAC-C-based deletion mutants with parental virus in CEF. Growth kinetics of parental and deletion mutants were similar (Figure 1C and Figure 2C), which indicated that the deleted genes are not necessary for virus replication in cultured CEF.

### 3.2. Cellular Immune Profile Induced by NYVAC-C Recombinants after Combined Deletion of VACV Immunomodulatory Genes

To define the effect of the distinct viral gene deletions in NYVAC-C vector immunogenicity, we used a mouse immunization protocol based on a DNA prime/poxvirus boost regime. This protocol is more immunogenic in activating T cell responses to HIV-1 antigens than the homologous combination of viral vectors and is being tested in clinical trials [8,11]. BALB/c mice (4 per group) were immunized as described (see Section 2). Memory T cell responses were analysed by polychromatic ICS assay 53 days after the last immunization. HIV-1-specific responses were measured after splenocyte stimulation with a panel of 464 peptides (15-mers overlapping by 11 amino acids) grouped in three pools (Env, 112 peptides; Gag, 121 peptides; and GPN, 231 peptides); vector-specific responses were detected using the VACV E3_140–148_ peptide. The percentages of T cells that produced IFN-γ and/or IL-2 and/or TNF-α indicated overall CD4^+^ and CD8^+^ T cell responses. The phenotype of the vaccine-induced memory responses was determined according to CD44 and CD62L surface marker expression on activated T cells (naïve, CD44^−^CD62L^+^; T central memory, TCM, CD44^+^CD62L^+^; T effector memory, EM, CD44^+^CD62L^−^; T terminal effector memory, TEM, CD44^−^CD62L^−^). Mice primed with sham DNA (DNA-ϕ) and boosted with non-recombinant NYVAC-WT were used as controls. A head-to-head comparison of immunogenicity among different NYVAC vectors is described below.

#### 3.2.1. Immunogenicity of NYVAC-C Recombinants Induced by Combined Deletion of VACV-TLR Inhibitors A46R, A52R, K7R and B15R

**Figure 3:**
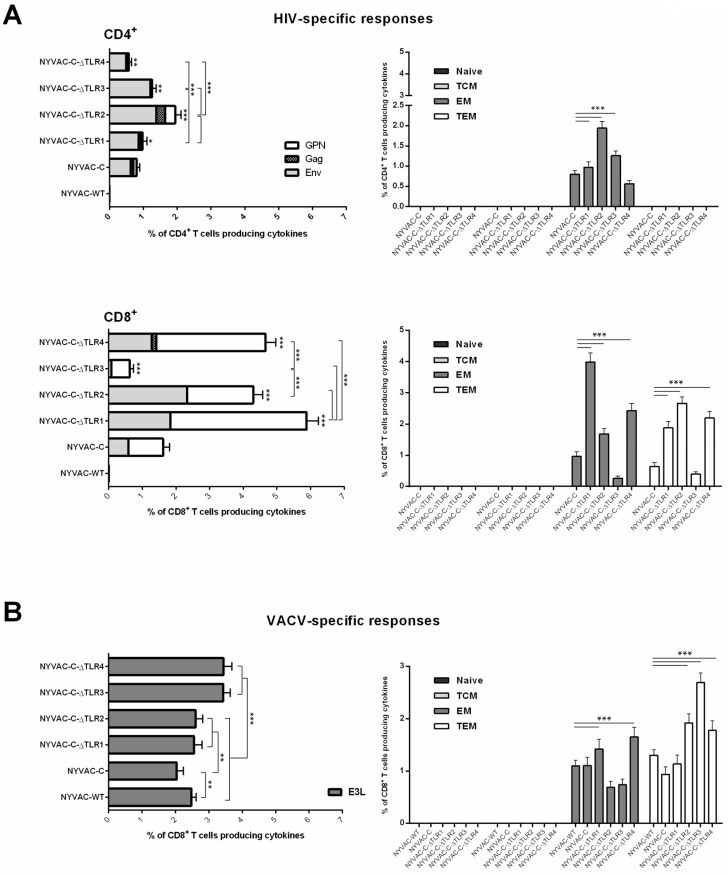
Cellular immune response elicited by NYVAC-C recombinant after sequential deletion of VACV-TLR inhibitors *A46R*, *A52R*, *K7R* and *B15R*. Magnitude and phenotypic profiles of (**A**) memory HIV-1-specific CD4^+^ (top) and CD8^+^ (bottom) T cells and (**B**) memory VACV-specific CD8^+^ T cells. The vaccine-induced cellular immune response was characterized by multi-parameter flow cytometry at 53 days after the last immunization. Values indicate the sum of the percentages of T cells that secrete IFN-γ and/or TNF-α and/or IL-2 in response to Env plus Gag plus GPN peptide pools (for HIV-1-specific responses) or to E3 peptide (for VACV-specific responses). Background percentages were subtracted from all data. The phenotype of the vaccine-induced memory responses was determined based on expression of CD44 and CD62L surface markers on activated T cells as follows: Naïve (CD44^−^CD62L^+^), T central memory (TCM; CD44^+^CD62L^+^), T effector memory (EM; CD44^+^CD62L^−^) or T terminal effector memory (TEM; CD44^−^CD62L^−^). * *p* < 0.05; ** *p* < 0.005, *** *p* < 0.001. Significant differences compared to the NYVAC-C group (for HIV-1 response) are indicated above each column.

Deletion of the VACV gene *A46R*, which encodes an inhibitor of TLR signalling, enhances immunogenicity of the NYVAC-C recombinant virus [15]. To define whether combined deletion of additional VACV-TLR inhibitors modulates the HIV-1- and VACV-specific immune responses elicited by NYVAC-C, we sequentially deleted *A52R*, *K7R* and *B15R* genes from a recombinant virus that already lacked *A46R* (NYVAC-C-∆TLR1), to generate deletion mutants NYVAC-C-∆TLR2 (∆A46R/∆A52R), NYVAC-C-∆TLR3 (∆A46R/∆A52R/∆K7R) and NYVAC-C-∆TLR4 (∆A46R/∆A52R/∆K7R/∆B15R). In DNA-C-primed mice boosted with the different viruses, the breadth of the HIV-1-specific responses was similar in all groups. CD4^+^ T cell responses were directed mainly against the Env pool, whereas CD8^+^ T cell responses were distributed between Env and GPN pools. With the exception of NYVAC-C-∆TLR3 virus, the deletion mutants triggered an HIV-1-specific memory response mediated mainly by CD8^+^ T cells (Figure 3A).

Compared to parental NYVAC-C, the single (NYVAC-C-∆TLR1), double (NYVAC-C-∆TLR2) and triple (NYVAC-C-∆TLR3) gene deletion mutants induced greater magnitudes of CD4^+^ T cell responses, with 1.2-, 2.45- and 1.6-fold increases, respectively. In contrast, the NYVAC-C-∆TLR4 virus elicited fewer HIV-specific CD4 T cells. The highest response was observed after sequential deletion of *A46R* and *A52R* genes (NYVAC-C-∆TLR2); single deletion of *A46R* or additional combined deletions (*K7R* and *B15R*) did not improve this response. The HIV-1-specific memory CD4^+^ T cells elicited by NYVAC-C and the deletion mutants had mainly an effector memory (EM) phenotype (Figure 3A, right).

For CD8^+^ T cells, the NYVAC-C-∆TLR1, -∆TLR2 and -∆TLR4 deletion mutants induced significantly larger HIV-1-specific responses than parental NYVAC-C, with respective increases of 3.7-, 2.7- and 2.9-fold. In this cell subset, single deletion of the *A46R* gene yielded the highest HIV-1-specific CD8^+^ T cell response; additional deletions did not increase these values. Deletion of the *K7R* gene in NYVAC-C-∆TLR2 (NYVAC-C-∆TLR3) reduced CD8^+^ T cell responses, which recovered after *B15R* deletion (NYVAC-C-∆TLR4). In all groups, the HIV-1-specific memory CD8 T cells elicited were distributed between the EM and TEM phenotypes. For parental NYVAC-C, distribution of the response was 60% _EM_/40% _TEM_. This ratio was maintained in the NYVAC-C-∆TLR1 group but was reversed to 40% _EM_/60% _TEM_ in NYVAC-C-∆TLR2 and NYVAC-C-∆TLR3 mutants. For NYVAC-C-∆TLR4, the response was distributed almost equally between the two phenotypes.

We also assessed the effect of these deletions on generation of VACV vector-specific responses, using the immunogenic VACV E3 protein as a marker. Compared to wild-type NYVAC virus, only NYVAC-C-∆TLR3 and NYVAC-C-∆TLR4 mutants showed increased anti-E3 CD8^+^ T cell responses, by about 1.5-fold. All deletion mutants nonetheless induced higher anti-VACV responses than parental NYVAC-C (Figure 3B). As for the HIV-1-specific response, the phenotypic profile of the E3-specific memory CD8^+^ T cells elicited by all viruses was distributed between EM and TEM phenotypes. For the NYVAC-C-∆TLR2 and -∆TLR3 groups, distribution of the response was 25% _EM_/75% _TEM_. For wild-type NYVAC virus, NYVAC-C, NYVAC-C-∆TLR1 and -∆TLR4, the response was distributed almost equally between the two phenotypes.

Except for NYVAC-C-∆TLR3, the remainder of the gene deletion mutants thus improved the HIV immunogenicity of parental NYVAC-C, with enhanced memory cell HIV-1-specific responses by the single (NYVAC-C-∆TLR1) and double (NYVAC-C-∆TLR2) mutants. For anti-vector immunity, the sequential removal of three or four VACV-TLR inhibitors was necessary to enhance responses.

#### 3.2.2. Effect of Combined Deletion of Several Unknown Non-Essential Genes and Cytokine Inhibitors on NYVAC-C Recombinant Immunogenicity

Since deletion of VACV genes that block IFN type I and II pathways improves the immunogenicity of NYVAC-C recombinants in mice [14], we explored whether combined deletion of several unknown, non-essential genes (*B6R*, *B9R* and *B10R*) with IFN—(*B8R* and *B19R*) and cytokine—(*B7R*) VACV inhibitors increased NYVAC-C immunogenicity. We deleted the *B6R-B10R* ORF cassette from a recombinant virus that lacked *B19R* (NYVAC-C-∆B19R), to generate NYVAC-C-∆B19R/∆B6R-B10R. Using the DNA prime/poxvirus boost approach, we evaluated the immune response induced by the resulting virus compared with parental NYVAC-C and with the single (NYVAC-C-∆B19R) and double (NYVAC-C-∆B8R/∆B19R) mutants.

**Figure 4:**
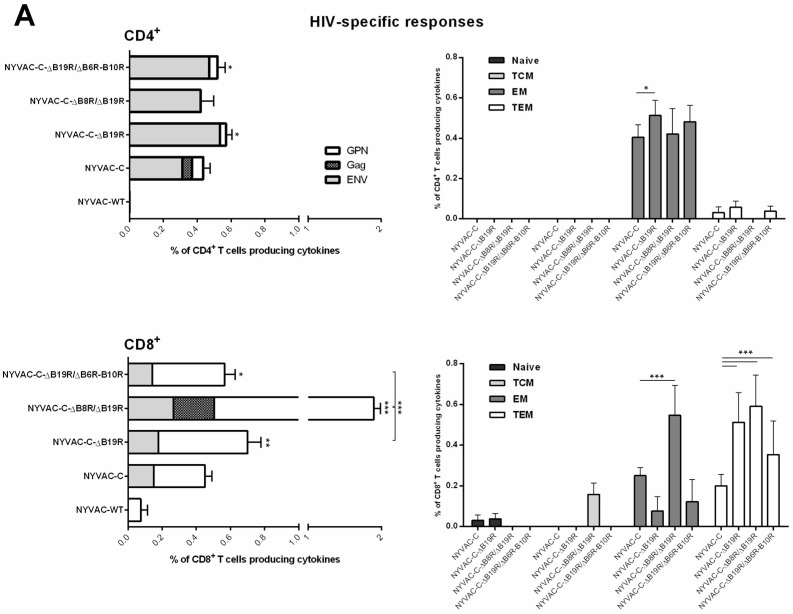

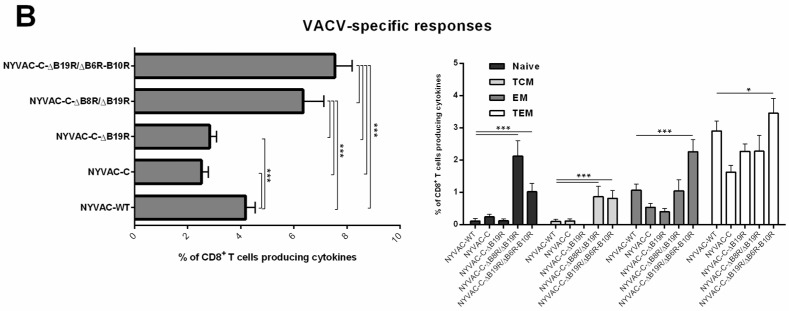
Cellular immune response elicited by NYVAC-C recombinant after combined deletion of various unknown non-essential genes with VACV-IFN and cytokine inhibitors. Magnitude and phenotypic profiles of (**A**) memory HIV-1-specific CD4^+^ (top) and CD8^+^ (bottom) T cells and (**B**) memory VACV-specific CD8^+^ T cells. The vaccine-induced cellular immune response was characterized by multi-parameter flow cytometry 53 days after the last immunization. Values indicate the sum of the percentages of T cells secreting IFN-γ and/or TNF-α and/or IL-2 against Env plus Gag plus GPN peptide pools (for HIV-1-specific responses) or E3 peptide (for VACV-specific responses). Background percentages were subtracted from all data. The phenotype of vaccine-induced memory responses was determined based on expression of CD44 and CD62L surface markers on activated T cells as in Figure 3. * *p* < 0.05; ** *p* < 0.005, *** *p* < 0.001. Significant differences compared to the NYVAC-C group (for HIV-1 response) are indicated above each column.

Compared to parental NYVAC-C, the NYVAC-C-∆B19R and NYVAC-C-∆B19R/∆B6R-B10R mutants induced modest increases in the magnitude of CD4^+^ T cell responses (~1.2-fold), while values for NYVAC-C-∆B8R/∆B19R virus were similar to NYVAC-C (Figure 4A). The HIV-1-specific memory CD4^+^ T cells elicited by NYVAC-C and the deletion mutants showed mainly an EM phenotype.

For CD8^+^ T cells, all deletion mutants induced higher HIV-1-specific responses than parental NYVAC-C, with increases of 1.5-fold for NYVAC-C-∆B19R, 2.9-fold for NYVAC-C-∆B8R/∆B19R and 1.2-fold for NYVAC-C-∆B19R/∆B6R-B10R. Double deletion of VACV-IFN inhibitors induced the highest antigen-specific CD8^+^ T cell responses; further combined deletion of unknown non-essential genes (*B6R*-*B10R*) and *B19R* cytokine inhibitor did not alter these values. In all groups, the HIV-1-specific memory CD8^+^ T cells were distributed mainly between the EM and TEM phenotypes. After NYVAC-C boost, approximately 60% and 40% of the HIV-1-specific response were EM and TEM, respectively, whereas the ratio in the NYVAC-C-∆B19R and NYVAC-C-∆B19R/∆B6R-B10R groups was 25% _EM_/75% _TEM_. For NYVAC-C-∆B8R/∆B19R, the response was distributed almost equally between the two phenotypes.

When we evaluated anti-VACV vector immunity compared to wild-type NYVAC virus, we found that E3-specific CD8^+^ T cell responses were enhanced in NYVAC-C-∆B8R/∆B19R and NYVAC-C-∆B19R/∆B6R-B10R groups, with ~2-fold increases, whereas for parental NYVAC-C and the single-gene deletion mutant NYVAC-C-∆B19R, VACV-specific responses were of similar magnitude (Figure 4B). E3-specific memory CD8^+^ T cells were distributed mainly between EM and TEM phenotypes, although some responses induced by NYVAC-C-∆B8R/∆B19R and NYVAC-C-∆B19R/∆B6R-B10R mutants had naïve and TCM phenotypes.

Combined deletion of *B6R*-*B10R* genes with the IFN inhibitor *B19R*, thus increased immunogenicity of parental NYVAC-C but did not modify the HIV-1-specific cellular responses elicited by the single and double gene deletion mutants that lacked the IFN inhibitors *B8R/B19R*; the deletions nonetheless significantly improved VACV-specific memory responses.

### 3.3. Combined Deletion of Immunomodulatory Genes Alters Humoral Responses to Env and VACV Vector

**Figure 5:**
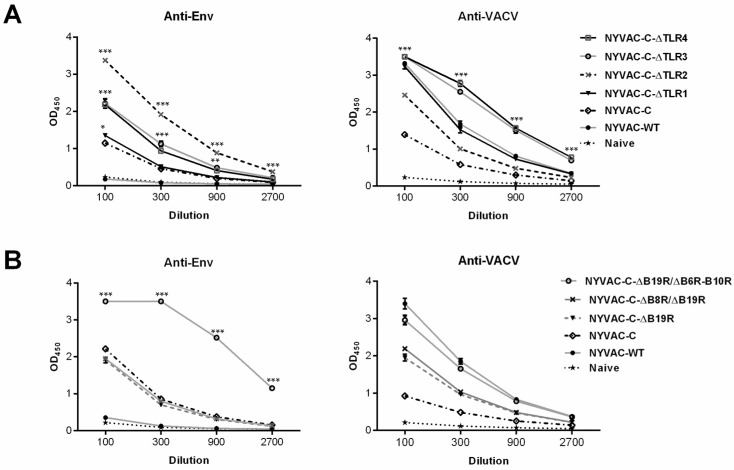
Anti-Env and -VACV humoral responses induced by NYVAC-C recombinants after combined deletion of VACV immunomodulatory genes. (**A**) Humoral responses after sequential deletion of VACV-TLR inhibitors *A46R*, *A52R*, *K7R* and *B15R*. (**B**) Humoral responses after deletion of several unknown non-essential genes in combination with VACV-IFN and cytokine inhibitors. ELISA assessment of levels of IgG antibodies to Env and vaccinia virus proteins in serum from naïve and immunized mice. Data shown as mean OD_450_ ± SD for each group at the dilutions assayed. * *p* < 0.05, ** *p* < 0.005, *** *p* < 0.001. Significant differences compared to the NYVAC-C group (anti-Env response, left) or the NYVAC-WT group (anti-VACV, right).

Since cells infected with NYVAC-C recombinants release monomeric gp120 [8], we evaluated how combined deletion of VACV immunomodulatory genes affected humoral responses at the memory phase. In ELISA, we quantified the Env- and VACV-specific IgG using recombinant CN54gp120 purified protein and cell extract from BSC-40 cells infected with VACV-WR strain, respectively (Figure 5).

Compared to parental NYVAC-C, the deletion mutants NYVAC-C-∆TLR2, NYVAC-C-∆TLR3 and NYVAC-C-∆TLR4 elicited higher levels of anti-Env antibodies at the dilutions tested (Figure 5A, left). Single deletion of the *A46R* gene (NYVAC-C-∆TLR1) only increased the anti-Env response at low serum dilutions (1:100). The highest antibody response was observed after sequential deletion of *A46R* and *A52R* genes (NYVAC-C-∆TLR2). We found that only NYVAC-C-∆TLR3 and NYVAC-C-∆TLR4 mutants improved the humoral anti-vector response induced by wild-type NYVAC virus, although the deletion mutants elicited higher anti-VACV antibody levels than parental NYVAC-C (Figure 5A, right).

In the group of viruses based on NYVAC-C after combined deletion of *B6R*-*B10R* with B19R, or the IFN inhibitors *B19R/B8R,* only the NYVAC-C-∆B19R/∆B6R-B10R mutant significantly enhanced the humoral Env-specific response compared with parental NYVAC-C. Single or double deletion of VACV-IFN inhibitors had no effect on the NYVAC-C-induced HIV-1 humoral immune response (Figure 5B, left). Although all the deletion mutants induced higher anti-VACV antibody levels than parental NYVAC-C, they did not improve the humoral anti-vector response induced by wild-type NYVAC (Figure 5B, right).

### 3.4. Single and Combined Deletion of VACV-TLR Inhibitors A46R, A52R, K7R and B15R Induced Similar “In Vivo” Protection against an Intranasal Challenge with a Lethal Dose of Wild-Type VACV WR

**Figure 6:**
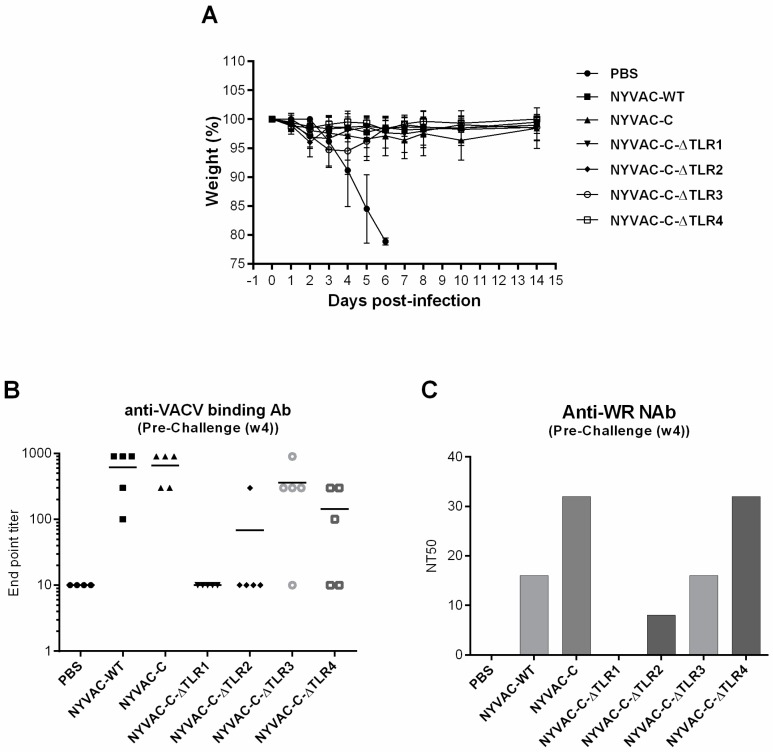
Vaccine efficacy of recombinant NYVAC-C viruses after single or sequential deletion of VACV-TLR inhibitors *A46R*, *A52R*, *K7R* and *B15R*. (**A**) Groups of five BALB/c mice were vaccinated by i.p. inoculation with 10^7^ pfu of the indicated viruses and challenged i.n. one month later with 5 × 10^6^ pfu of wild-type VACV WR. The resulting weight change was monitored daily. Data are expressed as the percentage ± standard error of the mean (SEM) of the mean weight of the same group of animals on day 0. (**B**) VACV-specific antibody titres and (**C**) VACV-specific neutralizing titres measured by ELISA and plaque reduction neutralization, respectively, in serum from immunized mice before the i.n. challenge.

To determine to what extent single and sequential gene deletion of VACV-TLR inhibitors impact on the vaccine efficacy compared with the NYVAC-C parental virus, mice were vaccinated i.p. and challenged i.n. one month later with a lethal dose of wild-type VACV WR. As shown in Figure 6A, all the viruses induced protection against challenge, indicated by similar weight loss over a period of 15 days compared with the PBS control group. However, before the intranasal challenge the levels of both the binding VACV-specific antibodies (Figure 6B) and the titre of neutralizing serum antibodies (Figure 6C) were different between the groups of vaccinated mice. Single deletion of the *A46R* gene (NYVAC-C-∆TLR1) and double deletion of *A46R* and *A52R* genes (NYVAC-C-∆TLR2) induced the lowest anti-vector humoral and neutralizing responses after a single i.p. immunization; however, animals were protected. 

## 4. Discussion

Given the modest efficacy observed in the RV144 clinical trial, the scientific community has focused on generating and optimizing vaccine candidates with improved immunogenicity, able to confer higher protection.

Poxviruses and particularly the highly attenuated VACV strains such as MVA and NYVAC, are being widely tested for potential HIV vaccines and are components in some of the clinical trials planned in the next few years [32,33,34] (https://clinicaltrials.gov/). Despite the safety and immunogenicity profiles of these attenuated VACV strains, it would be desirable to develop more efficient vectors that enhance the magnitude, breadth, polyfunctionality and durability of the T and B cell immune responses to exogenously expressed antigens. Various strategies are being used to achieve this purpose. One is deletion of viral immunomodulatory genes still present in the vector genome, whose products are predicted to interfere with optimal induction of cellular and humoral immune responses to vector-expressed antigens. We previously reported enhanced immunogenicity of MVA- and NYVAC-based recombinants with single, double or multiple deletions of VACV immunomodulatory genes such as *C12L* [35], *C6L* and/or *K7R* [36,37], *A41L* and/or *B16R* [30], *F1L* [38], *B8R* and/or *B19R* [14], *A46R* [15] or *A52R*, *K7R* and *B15R* in combination [16,17].

Here we extended these findings and explored the extent possibly improving NYVAC-C recombinant immunogenicity after combined deletion of genes involved in inhibition of TLR, IFN and cytokine/chemokine host-cell antiviral pathways or of genes with unknown immune function. We generated a collection of NYVAC-C deletion mutants, all of which expressed Env (gp120) and the HIV-1 clade C polyprotein Gag-Pol-Nef and tested them in mice for their immunogenic characteristics (CD4^+^/CD8^+^ T cells and antibodies) in response to HIV antigens and to the VACV vector (Table 1).

We analysed NYVAC-C recombinant immunogenicity after sequential deletion of the VACV-TLR inhibitors *A46R*, *A52R*, *K7R* and *B15R*. Double deletion of *A46R* and *A52R* was the best combination for enhancing cellular and humoral HIV-1-specific responses and increasing the percentage of CD8^+^ T cells with the TEM phenotype. Additional deletion of *K7R* and *B15R* did not further enhance the magnitude or quality of HIV-1-specific responses but significantly improved anti-vector immune responses.

We also examined whether deletion of various unknown non-essential genes in combination with VACV-IFN and -cytokine inhibitors enhanced NYVAC-C immunogenicity. The NYVAC-C-∆B19R/∆B6R-B10R deletion mutant significantly improved the HIV-1-specific humoral response but did not augment the HIV-1-specific cellular responses elicited by single or double gene deletion mutants lacking VACV-IFN inhibitors. This mutant significantly increased cellular and humoral VACV-specific memory responses. When we analysed in immunized mice the protection induced by the vectors lacking TLR inhibitors following an intranasal challenge with a lethal dose of wild-type VACV WR, we observed similar levels of protection between the vectors, with differences in total binding antibodies and neutralizing VACV titres. These findings highlight that deletions do not reduce the protective efficacy of the VACV-TLR inhibitors but influenced humoral responses.

Our overall analysis of the immunogenicity profiles induced by the NYVAC-C recombinants with selected deletions is summarized in Table 3, in which each NYVAC vector is ranked by levels of immune T and B cell activation compared with parental NYVAC-C. These results indicate that combined deletion of VACV immunomodulatory genes is a valuable strategy for improving immunogenicity of NYVAC-based vaccine candidates. The specific combination of gene deletions allows differential control of an immune response towards antigen- or vector-specific cellular and humoral responses. Deletion of more immunomodulatory genes from the NYVAC-C genome did not always guarantee more a robust anti-HIV-1 immune response, although some of these genes were necessary to improve VACV-specific responses. 

These results reflect the complexity and unpredictability of virus-host interactions in the context of an attenuated strain such as NYVAC.

Other groups have analysed the effect of multiple deletions in immunomodulatory genes on immune responses in the context of another attenuated poxvirus strain, MVA. Garber et al. assayed the effect on antigen-specific immune responses of simultaneous deletion of *C12L*, *B15R*, *A41L* and *A46R* genes, alone (MVA∆4) or combined with deletion of an essential viral replication gene (*udg*) (MVA∆5) in the genome of a recombinant MVA vector that expressed HIV *gag* and *env* genes [39]. Following a homologous prime-boost combination in rhesus macaques, they observed that both modified vectors significantly increase cellular and humoral HIV-specific immune responses compared to the control virus. Removal of the *udg* gene did not further improve HIV responses, however and offset the enhancement of vector-specific antibody titres due to immunization with the parental virus (∆4). Holgado et al. similarly reported that simultaneous deletion of *A44L*, *A46R* and *C12L* genes improved both innate and adaptive VACV-specific T cell immune responses in immunized mice, although the effect of these deletions in immunogenicity for a heterologous antigen was not tested [40]. Using MVA-BAC technology to examine the effect of deleting a gene cluster on immunogenicity of MVA deletion mutants, Alharbi et al. reported that none of the derived MVA vectors improved immunogenicity to MVA antigens or to the encoded heterologous antigen; this suggests that this approach should be assessed carefully for each recombinant antigen and epitope, rather than being used generically [41].

Although the results for MVA could not be extrapolated to NYVAC because of differences in vector genomes, it seems clear that combined deletion of selected immunomodulatory genes in the NYVAC genome should be considered for the design of viral recombinants as vaccine candidates.

## Figures and Tables

**Table 1:** New York vaccinia virus (NYVAC) deletion mutants used in this study. The genes are named according to Copenhagen strain nomenclature.

NYVAC Viruses	Deleted Gene/Function	Generation (Infection/Transfection Protocol)	
		Parental Virus	Plasmid Transfer Vector
NYVAC-WT	-	Not recombinant virus. Used as a control	
NYVAC-C	-	NYVAC recombinant virus expressing HIV-1 Env and Gag-Pol-Nef (clade C) antigens from the TK locus [8]. Used as a control for HIV responses	
NYVAC-C-∆TLR1	*A46R*/TLR signalling (NF-κB/IRF3) inhibitor [20]	[15]	
NYVAC-C-∆TLR2	*A46R*; *A52R*/TLR signalling (NF-κB) inhibitor [21]	NYVAC-C-∆TLR1	pGem-RG-A52R-wm
NYVAC-C-∆TLR3	*A46R*; *A52R*; *K7R*/TLR signalling (NF-κB/IRF3) inhibitor [22], promotes histone methylation [23]	NYVAC-C-∆TLR2	pGem-RG-K7R-wm
NYVAC-C-∆TLR4	*A46R*; *A52R*; *K7R*; *B15R*/TLR signalling (IkappaB kinase) inhibitor [24]	NYVAC-C-∆TLR3	pGem-RG-B15R-wm
NYVAC-C-∆B19R	*B19R*/IFN-α/β soluble receptor [25]	[18]	
NYVAC-C-∆TLR4/∆B19R	*A46R*; *A52R*; *K7R*; *B15R*; *B19R*	NYVAC-C-∆TLR4	pGem-RG-B19R-wm [18]
NYVAC-C-∆B8R/∆B19R	*B8R*/IFN-γ soluble receptor [26]; *B19R*	[14]	
NYVAC-C-∆B19R/∆B6R-B10R	*B6R*/unknown; *B7R*/TNF-α soluble receptor and chemokine binding protein [27]; *B8R*; *B9R*/intracellular protein [28]; *B10R*/unknown; *B19R*	NYVAC-C-∆B19R	pGem-RG-B6R-B10R-wm

TK: Thymidine kinase locus; TLR: Toll-like receptor; NF-κB: Nuclear factor kappa B; IRF3: Interferon regulatory factor 3; IFN: Interferon; TNF: Tumor necrosis factor.

**Table 2:** Primers used for the deletion/confirmation of deletion by PCR of *A46R*, *A52R*, *K7R*, *B15R*, *B8R*, *B19R* and *B6R-B10R* open reading frames (ORFs). Restriction enzymes cleavage sites are underlined.

Locus	Sequence of Primers (5′→3′)	Size of Amplified Product (bp)	
		Parental Virus	Deletion Mutant
*A46R*	LFA46R-Aat: CACGATGACGTCAGAGGAGTTAT RFA46R-Bam: ATTTAAGGATCCAGAACGGCAAC	1342	774
*A52R*	LF′A52R-Eco: ATTAGAGAATTCTACGATTAACGA RFA52R-Bam: TCTGCCGGATCCAATGTAGTAATG	1320	747
*K7R*	K7R-F: TATGATCATGTGAGAATACTAAAATTCC K7R-R: CCGAATTGGGTAGACGATGTATGAATCC	1048	626
*B15R*	LF′B15R-Aat: TTCTTTGACGTCTGTTTTCCTGAAG RFB15R-Bam: GTGTCGGGATCCGAATTAGCATATT	1128	701
*B8R*	LFB8R-AatII-F: TTTTTTGACGTCATTGACTCGTCTACTATTC RFB8R-BamHI-R: TTTTTTGGATCCAAACAGCGGACACATTGC	1519	706
*B19R*	LFB19R-AatII-F: TTTTTTGACGTCGAGAAAGTTAAGAAGATAC RFB19R-BamHI-R: TTTTTTGGATCCAGTTCTATCATAATCATC	1782	726
*B6R-B10R*	LFB6R-AatII-F: GGAATGACGTCCTCCCAATATGTG RFB10R-BamHI-R: CGGGATCCAGTAGATATGATCTATATTC	3283	725

**Table 3:** T cell phenotype and antibody responses induced by the different NYVAC deletion mutants.

NYVAC Deletion Mutant	HIV-Specific Response					VACV-Specific Response			
	CD4 T Cells	CD8 T Cells	Memory Phenotype		BAbs	CD8 T Cells	Memory CD8 T Cells Phenotype	BAbs	NAbs
			CD4 T Cells	CD8 T Cells					
NYVAC-C-∆TLR1	+	+++	EM	EM > TEM	+	+	EM ≥ TEM	++	+
NYVAC-C-∆TLR2	+++	++	EM	EM < TEM	+++	+	EM < TEM	+	=
NYVAC-C-∆TLR3	++	−	EM	EM < TEM	++	++	EM < TEM	+++	+++
NYVAC-C-∆TLR4	−	++	EM	EM ≥ TEM	++	++	EM ≤ TEM	+++	++
NYVAC-C-∆B19R	+	++	EM	EM < TEM	=	=	EM < TEM	++	+
NYVAC-C-∆B8R/∆B19R	=	++++	EM	EM < TEM	=	+++	EM ≤ TEM	++	+
NYVAC-C-∆B19R/∆B6R-B10R	+	+	EM	EM < TEM	+++	++++	EM < TEM	+++	+

BAbs: Binding antibodies; NAbs: Neutralizing antibodies; EM: Effector memory; TEM: Terminal effector memory. The symbols “+”, “−” or “=” indicate an increase, decrease or no effect in the immune response elicited compared to the parental NYVAC-C.
